# Hand-Gesture Recognition Based on EMG and Event-Based Camera Sensor Fusion: A Benchmark in Neuromorphic Computing

**DOI:** 10.3389/fnins.2020.00637

**Published:** 2020-08-05

**Authors:** Enea Ceolini, Charlotte Frenkel, Sumit Bam Shrestha, Gemma Taverni, Lyes Khacef, Melika Payvand, Elisa Donati

**Affiliations:** ^1^Institute of Neuroinformatics, University of Zurich, ETH Zurich, Zurich, Switzerland; ^2^ICTEAM Institute, Université Catholique de Louvain, Louvain-la-Neuve, Belgium; ^3^Temasek Laboratories, National University of Singapore, Singapore, Singapore; ^4^Université Côte d'Azur, CNRS, LEAT, Nice, France

**Keywords:** hand-gesture classification, spiking neural networks (SNNs), electromyography (EMG) signal processing, event-based camera, sensor fusion, neuromorphic engineering

## Abstract

Hand gestures are a form of non-verbal communication used by individuals in conjunction with speech to communicate. Nowadays, with the increasing use of technology, hand-gesture recognition is considered to be an important aspect of Human-Machine Interaction (HMI), allowing the machine to capture and interpret the user's intent and to respond accordingly. The ability to discriminate between human gestures can help in several applications, such as assisted living, healthcare, neuro-rehabilitation, and sports. Recently, multi-sensor data fusion mechanisms have been investigated to improve discrimination accuracy. In this paper, we present a sensor fusion framework that integrates complementary systems: the electromyography (EMG) signal from muscles and visual information. This multi-sensor approach, while improving accuracy and robustness, introduces the disadvantage of high computational cost, which grows exponentially with the number of sensors and the number of measurements. Furthermore, this huge amount of data to process can affect the classification latency which can be crucial in real-case scenarios, such as prosthetic control. Neuromorphic technologies can be deployed to overcome these limitations since they allow real-time processing in parallel at low power consumption. In this paper, we present a fully neuromorphic sensor fusion approach for hand-gesture recognition comprised of an event-based vision sensor and three different neuromorphic processors. In particular, we used the event-based camera, called DVS, and two neuromorphic platforms, Loihi and ODIN + MorphIC. The EMG signals were recorded using traditional electrodes and then converted into spikes to be fed into the chips. We collected a dataset of five gestures from sign language where visual and electromyography signals are synchronized. We compared a fully neuromorphic approach to a baseline implemented using traditional machine learning approaches on a portable GPU system. According to the chip's constraints, we designed specific spiking neural networks (SNNs) for sensor fusion that showed classification accuracy comparable to the software baseline. These neuromorphic alternatives have increased inference time, between 20 and 40%, with respect to the GPU system but have a significantly smaller energy-delay product (EDP) which makes them between 30× and 600× more efficient. The proposed work represents a new benchmark that moves neuromorphic computing toward a real-world scenario.

## 1. Introduction

Hand-gestures are considered a powerful communication channel for information transfer in daily life. Hand-gesture recognition is the process of classifying meaningful gestures of the hands and is currently receiving renewed interest. The gestural interaction is a well-known technique that can be utilized in a vast array of applications (Yasen and Jusoh, 2019), such as sign language translation (Cheok et al., 2019), sports (Loss et al., 2012), Human-Robot Interaction (HRI) (Cicirelli et al., 2015; Liu and Wang, 2018), and more generally in Human-Machine Interaction (HMI) (Haria et al., 2017). Hand-gesture recognition systems also target medical applications, where they are detected via bioelectrical signals instead of vision. In particular, among the biomedical signals, electromyography [Electromyography (EMG)] is the most used for hand-gesture identification and for the design of prosthetic hand controllers (Benatti et al., 2015; Donati et al., 2019; Chen et al., 2020).

EMG measures the electrical signal resulting from muscle activation. The source of the signal is the motor neuron action potentials generated during the muscle contraction. Generally, EMG can be detected either directly with electrodes inserted in the muscle tissue, or indirectly with surface electrodes positioned above the skin [surface EMG (sEMG), for simplicity we will refer to it as EMG]. The EMG is more popular for its accessibility and non-invasive nature. However, the use of EMG to discriminate between hand-gestures is a non-trivial task due to several physiological processes in the skeletal muscles underlying their generation.

One way to overcome these limitations is to use a multimodal approach, combining EMG with recordings from other sensors. Multi-sensor data fusion is a direct consequence of the well-accepted paradigm that certain natural processes and phenomena are expressed under completely different physical guises (Lahat et al., 2015). In fact, multi-sensor systems provide higher accuracy by exploiting different sensors that measure the same signal in different but complementary ways. The higher accuracy is achieved thanks to a redundancy gain that reduces the amount of uncertainty in the resulting information. Recent works show a growing interest toward multi-sensory fusion in several application areas, such as developmental robotics (Droniou et al., 2015; Zahra and Navarro-Alarcon, 2019), audio-visual signal processing (Shivappa et al., 2010; Rivet et al., 2014), spatial perception (Pitti et al., 2012), attention-driven selection (Braun et al., 2019) and tracking (Zhao and Zeng, 2019), memory encoding (Tan et al., 2019), emotion recognition (Zhang et al., 2019), multi-sensory classification (Cholet et al., 2019), HMI (Turk, 2014), remote sensing and earth observation (Debes et al., 2014), medical diagnosis (Hoeks et al., 2011), and understanding brain functionality (Horwitz and Poeppel, 2002).

In this study we consider the complementary system comprising of a vision sensor and EMG measurements. Using EMG or camera systems separately presents some limitations, but their fusion has several advantages, in particular EMG-based classification can help in case of camera occlusion, whereas the vision classification provides an absolute measurement of hand state. This type of sensor fusion which combines vision and proprioceptive information is intensively used in biomedical applications, such as in the transradial prosthetic domain, to improve control performance (Markovic et al., 2014, 2015), or to focus on recognizing objects during grasping to adjust the movements (Došen et al., 2010). This last task can also use Convolutional Neural Networks (CNNs) as feature extractors (Ghazaei et al., 2017; Gigli et al., 2018).

While improving accuracy and robustness, the multiple input modalities also increase the computational cost, due to the amount of data generated to process in real-time which can affect the communication between the subject and the prosthetic hand. Neuromorphic technology offers a solution to overcome these limitations providing the possibility to process multiple inputs in parallel in real-time, and with very low power consumption. Neuromorphic systems consist of circuits designed with principles based on the biological nervous systems that, similar to their biological counterparts, process information using energy-efficient, asynchronous, event-driven methods (Liu et al., 2014). These systems are often endowed with on-line learning abilities that allow adapting to different inputs and conditions. Lots of neuromorphic computing platforms have been developed in the past for modeling cortical circuits and their number is still growing (Benjamin et al., 2014; Furber et al., 2014; Merolla et al., 2014; Meier, 2015; Qiao et al., 2015; Moradi et al., 2017; Davies et al., 2018; Neckar et al., 2018; Thakur et al., 2018; Frenkel et al., 2019a,b).

In this paper we present a fully-neuromorphic implementation of sensor fusion for hand-gesture recognition. The proposed work is based on a previous work of sensor fusion for hand-gesture recognition, using standard machine learning approaches implemented in a cell phone application for personalized medicine (Ceolini et al., 2019b). The paper showed how a CNN performed better, in terms of accuracy, than a Support Vector Machine (SVM) on the hand-gesture recognition task. The novelty introduced here is that the sensor fusion is implemented on a fully neuromorphic system, from the event-based camera sensor to the classification phase, performed using three event-based neuromorphic circuits: Intel's Loihi research processor (Davies et al., 2018) and a combination of the ODIN and MorphIC Spiking Neural Network (SNN) processors (Frenkel et al., 2019a,b). The two neuromorphic systems present different features, in particular, depending on the number of neurons available and on the input data, we implemented different SNN architectures. For example, for visual data processing, a spiking CNN is implemented in Loihi while a spiking Multi-Layer Perceptron (MLP) is chosen for ODIN + MorphIC (see section 2.3). For the case of EMG, the data was collected using the Myo armband that senses electrical activity in the forearm muscles. The data was later converted into spikes to be fed into the neuromorphic systems. Here, we propose a feasible application to show the neuromorphic performance in terms of accuracy, energy consumption, and latency (stimulus duration + inference time). The performance metric for the energy consumption is the Energy-Delay Product (EDP), a metric suitable for most modern processor platforms defined as the average energy consumption multiplied by the average inference time. The inference time is defined as the time elapsed between the end of the stimulus and the classification. To validate the neuromorphic results, we are comparing it to a baseline consisting of the network implemented, using a standard machine learning approach, where the inputs are fed as continuous EMG signals and video frames. We propose this comparison for a real case scenario as a benchmark, in order for the neuromorphic research field to advance into mainstream computing (Davies, 2019).

## 2. Materials and Methods

In the following section, we describe the overall system components. We start from the description of the sensors used to collect the hand-gesture data, namely the event-based camera, Dynamic Vision Sensor (DVS), and the EMG armband sensor, Myo. We then describe the procedure with which we collected the dataset used for the validation experiments presented here and which is publicly available. Afterwards, the two neuromorphic systems under consideration, namely Loihi and ODIN + MorphIC, will be described, focusing on their system specifics, characteristics, and the model architectures that will be implemented on them. Finally, we describe the system that we call baseline and which represents the point of comparison between a traditional von-Neumann approach and the two neuromorphic systems.

### 2.1. DVS and EMG Sensors

#### 2.1.1. DVS Sensor

**Figure 1:**
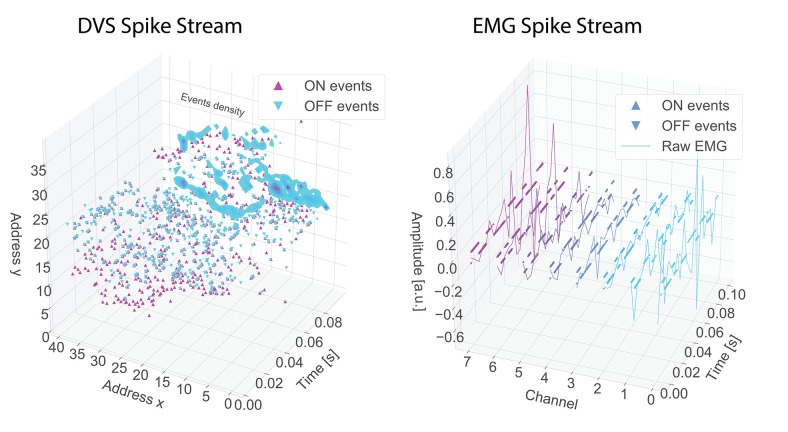
Example, for a gesture “elle,” of spike streams for DVS (left) and EMG (right). In the EMG figure the spikes are represented by dots while the continuous line is the raw EMG. Different channels have different colors.

The DVS (Lichtsteiner et al., 2006) is a neuromorphic camera inspired by the visual processing in the biological retina. Each pixel in the sensor array responds asynchronously to logarithmic changes in light. Whenever the incoming illumination increases or decreases above a certain threshold, it generates a polarity spike event. The polarity corresponds to the sign of the change; ON polarity for an increase in light, and OFF polarity for a decrease in light. The output is a continuous and sparse train of events, interchangeably called spikes throughout this paper, that carries the information of the active pixels in the scene (represented in Figure 1). The static information is directly removed on the hardware side and only the dynamic one, corresponding to the movements in the scene, is actually transmitted. In this way the DVS can reach low latency, down to 10 μs, reducing the power consumption needed for computation and the amount of transmitted data. Each spike is encoded using the Address Event Representation (AER) communication protocol (Deiss et al., 1999) and is represented by the address of the pixel (in x-y coordinates), the polarity (1 bit for the sign), and the timestamp (in microsecond resolution).

#### 2.1.2. EMG Sensor

In the proposed work, we collected the EMG corresponding to hand gestures using the Myo armband by Thalmic Labs Inc. The Myo armband is a wearable device provided with eight equally spaced non-invasive EMG electrodes and a Bluetooth transmission module. The EMG electrodes detect signals from the forearm muscles activity and afterwards the acquired data is sent to an external electronic device. The sampling rates for Myo data are fixed at 200 Hz and the data is returned as a unitless 8-bit unsigned integer for each sensor representing “activation” and does not translate to millivolts (mV).

### 2.2. DVS-EMG Dataset

**Figure 2:**
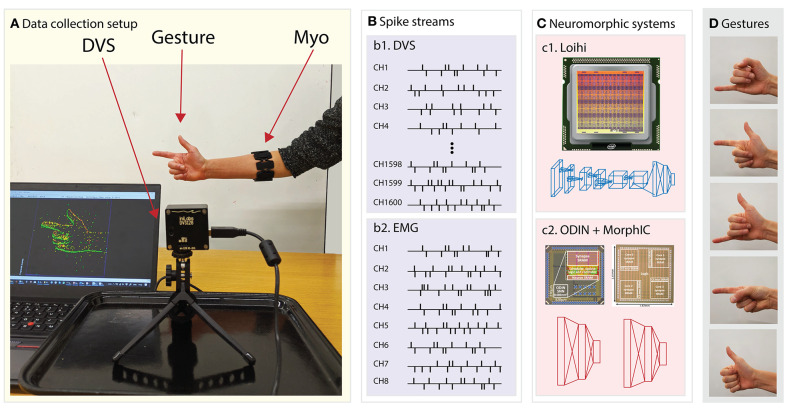
System overview. From left to right: **(A)** data collection setup featuring the DVS, the traditional camera and the subject wearing the EMG armband sensor, **(B)** data streams of (b1) DVS and (b2) EMG transformed into spikes via the Delta modulation approach, **(C)** the two neuromorphic systems namely (c1) Loihi and (c2) ODIN + MorphIC, **(D)** the hand gestures that the system is able to recognize in real time.

The dataset is a collection of five hand gestures recorded with the two sensor modalities: muscle activity from the Myo and visual input, in the form of DVS events. Moreover, the dataset also provides the video recording using a traditional frame-based camera, referred to as Active Pixel Sensor (APS) in this paper. The frames from the APS are used as ground truth and as input in the baseline models. The APS-frames provided in the dataset are gray-scale, 240 × 180 resolution. The dataset contains recordings from 21 subjects: 12 males and nine females aged from 25 to 35 (see Data Availability Statement for the full access to the dataset). The structure is the following: each subject repeats three sessions, in each session the subject performs five hand gestures: *pinky, elle, yo, index*, and *thumb* (see Figure 2), repeated 5 times. Each single gesture recording lasts 2*s*. The gestures are separated by a relaxing time of 1*s*, to remove any residual activity from the previous gesture. Every recording is cut in 10 chunks of 200*ms* each, this duration was selected to match the requirements of a real-case scenario of low latency prosthesis control where there is a need for the classification and creation of the motor command within 250 *ms* (Smith et al., 2011). Therefore, the final number of samples results in 21 (subjects) × 3 (trials) × 5 (repetitions) × 5 (gestures) × 10 (chunks) for a total of 15,750. The Myo records the superficial muscle activity at the middle forearm from eight electrodes with a sampling rate of 200*Hz*. During the recordings, the DVS was mounted on a random moving system to generate relative movement between the sensor and the subject's hand. The hand remains static during the recording to avoid noise in the Myo sensor and the gestures are performed in front of a static white background, see Figure 2 for the full setup.

#### 2.2.1. Implementation on Neuromorphic Devices

SNNs, in general, and their implementation on neuromorphic devices require inputs as spike trains. In the case of the DVS, the sensor output is already in the form of spikes and polarity. The only requirement that we need to take into account is the limited number of neurons in the available neuromorphic processors. For this reason, we decided to crop the 128 × 128 input of the DVS to 40 × 40 centered on the hand-gesture. On the contrary, for the EMG, a conversion in the event-based domain is required. The solution used here is the delta-modulator ADC algorithm, based on a sigma-delta modulator circuit (Corradi and Indiveri, 2015). This mechanism is particularly used in low frequency, high performance and low power applications (Lee et al., 2005), such as biomedical circuits. Moreover, this modulator represents a good interface for neuromorphic devices because it has much less circuit complexity and lower power consumption than multi-bit ADCs.

The delta-modulator algorithm transforms a continuous signal into two digital pulse outputs, UP or DOWN, according to the signal derivative. The UP (DOWN) spikes are generated every time the signal exceeds a positive (negative) threshold, like the ON (OFF) events from the DVS. As described before, the signal is sampled at 200*Hz*, this means that a new sample is acquired every 5 *ms*. To increase the time resolution of the generated spike train, which otherwise would contain too few spikes, the EMG signals are over-sampled to a higher frequency before undergoing the transformation into spikes (Donati et al., 2019).

For our specific EMG acquisition features, we set the threshold at 0.05 and an interpolation factor of 3500; these values have been selected from previous studies which looked at quality of signal reconstruction (Donati et al., 2018, 2019).

### 2.3. Neuromorphic Processors

#### 2.3.1. ODIN + MorphIC

The ODIN (Online-learning DIgital spiking Neuromorphic) processor occupies an area of only 0.086 mm^2^ in 28 nm FDSOI CMOS (Frenkel et al., 2019a)^1^. It consists of a single neurosynaptic core with 256 neurons and 256^2^ synapses. Each neuron can be configured to phenomenologically reproduce the 20 Izhikevich behaviors of spiking neurons (Izhikevich, 2004). The synapses embed a 3-bit weight and a mapping table bit that allows enabling or disabling Spike-Dependent Synaptic Plasticity (SDSP) locally (Brader et al., 2007), thus allowing for the exploration of both off-chip training and on-chip online learning setups.

MorphIC is a quad-core digital neuromorphic processor with 2k LIF neurons and more than 2M synapses in 65nm CMOS (Frenkel et al., 2019b). MorphIC was designed for high-density large-scale integration of multi-chip setups. The four 512-neuron crossbar cores are connected with a hierarchical routing infrastructure that enables neuron fan-in and fan-out values of 1k and 2k, respectively. The synapses are binary and can be either programmed with offline-trained weights or trained online with a stochastic version of SDSP.

Both ODIN and MorphIC follow a standard synchronous digital implementation, which allows their operation to be predicted with one-to-one accuracy by custom Python-based chip simulators. As both chips rely on crossbar connectivity, CNN topologies can be explored but are limited to small networks due to an inefficient resource usage in the absence of a weight reuse mechanism (Frenkel et al., 2019b). The selected SNN architectures are thus based on fully-connected MLP topologies. Training is carried out in Keras with quantization-aware stochastic gradient descent following a standard ANN-to-SNN mapping approach (Hubara et al., 2017; Moons et al., 2017; Rueckauer et al., 2017), the resulting SNNs process the EMG and DVS spikes without further preprocessing.

In order to process the spike-based EMG gesture data, we selected ODIN so as to benefit from 3-bit weights. Indeed, due to the low input dimensionality of EMG data, satisfactory performance could not be reached with the binary weight resolution of MorphIC. A 3-bit-weight 16-230-5 SNN is thus implemented in ODIN, this setup will be referred to as the EMG-ODIN network.

For the DVS gesture data classification, we selected MorphIC, to benefit from its higher neuron and synapse resources. ON/OFF DVS events are treated equally and their connections to the network are learned, so that any of them can be either excitatory or inhibitory. Similarly to a setup previously proposed for MNIST benchmarking (Frenkel et al., 2019b), the input 40 × 40-pixel DVS event streams can be subsampled into four 20 × 20-pixel event streams and processed independently in the four cores of MorphIC, thus leading to an accuracy boost when combining the outputs of all subnetworks, subsequently denoted as subMLPs. The four subMLPs have a 400-210-5 topology with binary weights, this setup will thus be referred to as the DVS-MorphIC network.

To ease sensor fusion, the hidden layer sizes of the EMG-ODIN and DVS-MorphIC networks and the associated firing thresholds were optimized by parameter search so as to balance their activities. These hidden layers were first flattened into a 1,070-neuron layer, then a 5-neuron output layer was retrained with 3-bit weights and implemented in ODIN. This setup will be referred to as the Fusion-ODIN network, which thus encapsulates EMG processing in ODIN, DVS processing in MorphIC, and sensor fusion in ODIN. From an implementation point of view, mapping the MorphIC hidden layer output spikes back to ODIN as sensor fusion requires an external mapping table. Its overhead is excluded from the results provided in section 3.

#### 2.3.2. Loihi and Its Training Framework SLAYER

Intel's Loihi (Davies et al., 2018) is an asynchronous neuromorphic research processor. Each Loihi chip consists of 128 neurocores, with each neurocore capable of implementing up to 1,024 current based (CUBA) Leaky Integrate and Fire (LIF) neurons. The network state and configuration is stored entirely in on-chip SRAMs local to each core, this allows each core to access its local memories independently of other cores without needing to share a global memory bus (and in fact removing the need for off-chip memory). Loihi supports a number of different encodings for representing network connectivity, thus allowing the user to choose the most efficient encoding for their task. Each Loihi chip also contains three small synchronous ×86 processors which help monitor and configure the network, as well as assisting with the injection of spikes and recording of output spikes.

SLAYER (Shrestha and Orchard, 2018) is a backpropagation framework for evaluating the gradient of any kind of SNN [i.e., spiking MLP and spiking CNN] directly in the spiking domain. It is a dt-based SNN backpropagation algorithm that keeps track of the internal membrane potential of the spiking neuron and uses it during gradient propagation. There are two main guiding principles of SLAYER: temporal credit assignment policy and probabilistic spiking neuron behavior during error backpropagation. Temporal credit assignment policy acknowledges the temporal nature of a spiking neuron where a spike event at a particular time has its effect on future events. Therefore, the error credit of an error at a particular time needs to be distributed back in time. SLAYER is one of the few methods that consider temporal effects during backpropagation. The use of probabilistic neurons during backpropagation helps estimate the spike function derivative, which is a major challenge for SNN backpropagation, with the spike escape rate function of a probabilistic neuron. The end effect is that the spike escape rate function is used to estimate the spike function derivative, similar to the surrogate gradient concept (Zenke and Ganguli, 2018; Neftci et al., 2019). With SLAYER, we can train synaptic weights as well as axonal delays and achieve state of the art performances (Shrestha and Orchard, 2018) on neuromorphic datasets.

SLAYER uses the versatile Spike Response Model (SRM) (Gerstner, 1995) which can be customized to represent a wide variety of spiking neurons with a simple change of spike response kernels. It is implemented^2^ atop the PyTorch framework with automatic differentiation support (Paszke et al., 2017) with the flexibility of feedforward dense, convolutional, pooling, and skip connections in the network.

SLAYER-PyTorch also supports training with the exact CUBA Leaky Integrate and Fire neuron model in Loihi (Davies et al., 2018). To train for the fixed precision constraints on weights and delays of Loihi hardware, it trains the network with the quantization constraints and then trains using the strategy of shadow variables (Courbariaux et al., 2015; Hubara et al., 2016) where the constrained network is used in the forward propagation phase and the full precision shadow variables are used during backpropagation.

We used SLAYER-PyTorch to train a Loihi compatible network for the hand-gesture recognition task. The networks were trained offline using GPU and trained weights and delays were used to configure the network on Loihi hardware for inference purposes. All the figures reported here are for inference using Loihi, with one algorithmic time tick in Loihi of 1 *ms*.

A spiking MLP of architecture 16-128d-128d-5 was trained for EMG gestures converted into spikes (section 2.2.1). Here, 128d means the fully connected layer has 128 neurons with trained axonal delays. The Loihi neuron with current and voltage decay constants of 1,024 (32 ms) was used for this network.

For the gesture classification using DVS data we used both a spiking MLP, with the same architecture as the one deployed on MorphIC and described in section 2.3.1, and a spiking CNN with architecture 40x40x2-8c3-2p-16c3-2p-32c3-512-5. Here, XcY denotes a convolution layer with X kernels of shape Y-by-Y, while 2p denotes a 2-by-2 max pooling layer. Zero padding was applied for all convolution layers. No preprocessing on the spike events was performed, the ON/OFF events are treated as different input channels, hence the input shape 40x40x2. For this network, current and voltage decay constants for the Loihi neurons were set to 1,024 (32 ms) and 128 (4 ms).

Finally, a third network where the penultimate layer neurons of DVS and EMG networks were fused together was trained. Only the last fully connected weights (640-5) were trained. The parameters of the network before fusion were preserved. The current and voltage decay constants of 1,024 (32 ms) and 128 (4 ms), respectively, were used for the final fusion layer neurons. From now on, we will refer to these three networks as EMG-Loihi, DVS-Loihi, and Fusion-Loihi whenever there is ambiguity.

### 2.4. Traditional Machine Learning Baselines

**Figure 3:**
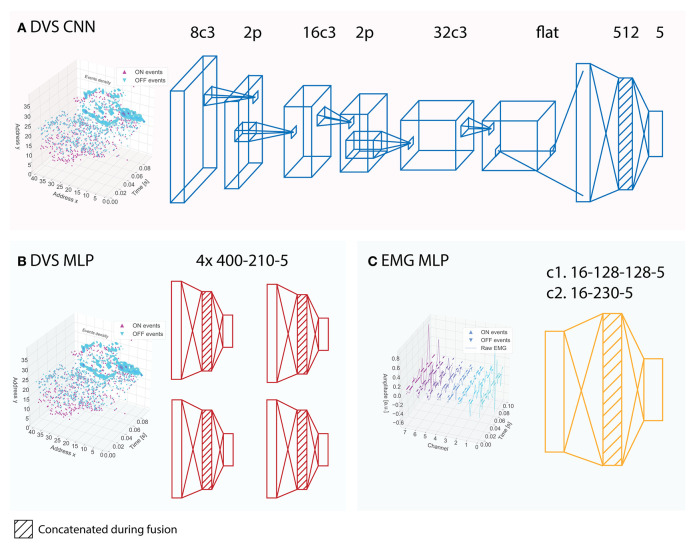
Architectures of the neural networks implemented on the neuromorphic systems and used in the baselines. **(A)** CNN architecture implemented on Loihi; the corresponding baseline CNN receives APS frames instead of DVS events. **(B)** subMLP architectures implemented on MorphIC, the corresponding baseline subMLPs receive APS frames instead of DVS events. **(C)** MLP architecture for the EMG data implemented on Loihi (c1) and on ODIN (c2), the corresponding baseline MLPs receive EMG features instead of EMG events. The shading indicates those layers that are concatenated during the fusion of the networks.

Machine Learning (ML) methods, and in general data-driven approaches, are currently the dominant tools used to solve complex classification tasks since they give the best performance compared to other approaches. We compare the performance of the two fully neuromorphic systems described in the above sections, against a traditional machine learning pipeline that uses frame-based inputs, i.e., traditionally sampled EMG signals and traditionally sampled video frames. For the comparisons to be fair, in the traditional approach we maintain the same constraints imposed by the neuromorphic hardware. In particular, we used the same neural network architectures as those used in the neuromorphic systems. Note that two different networks were implemented, spiking MLP and spiking CNN (see Figure 3 for more details on the architectures). For this reason, we have two different baseline models that are paired to the two considered neuromorphic systems.

#### 2.4.1. EMG Feature Extraction

Traditional EMG signal processing consists of various steps. First, signal pre-processing is used to extract useful information by applying filters and transformations. Then, feature extraction is used to highlight meaningful structures and patterns. Finally, a classifier maps the selected features to output classes. In this section we describe the EMG feature extraction phase, in particular we consider time domain features used for the classification of gestures with the baseline models. We extracted two time domain features generally used in literature (Phinyomark et al., 2018), namely Mean Absolute Value (MAV) and Root Mean Square (RMS) shown in Equation (1). The MAV is the average of the muscles activation value and it is calculated by a stride-moving window. The RMS is represented as amplitude relating to a gestural force and muscular contraction. The two features are calculated across a window of 40 samples, corresponding to 200 ms:

(1) $$\begin{array}{l} MAV\left(x_{c}\right)=\frac{1}{T}\overset{T}{\underset{t=0}{\sum }}|x_{c}\left(t\right)|\;RMS\left(x_{c}\right)=\sqrt{\frac{1}{T}\overset{T}{\underset{t=0}{\sum }}x_{c}^{2}\left(t\right)} \end{array}$$

where *x*_*c*_(*t*) is the signal in the time domain for the EMG channel with index *c* and *T* is the number of samples in the considered window, which was set to *T* = 40 (*N* = 200 ms) across this work. The features were calculated for each channel separately and the resulting values were concatenated in a vector **F**(*n*) described in Equation (2):

$$\begin{array}{l} \text{F}\left(n\right)={\left[F\left(x_{1}\right),\ldots ,F\left(x_{C}\right)\right]}^{T} \end{array}$$

where **F** is MAV or RMS, *n* is the index of the window and *C* is the number of EMG channels. The final feature vector **E**(*n*) for window *n* is shown in Equation (3), it is used for the classification and is obtained by concatenating the two single feature vectors.

$$\begin{array}{l} \text{E}\left(n\right)={\left[\text{MAV}{\left(n\right)}^{T},\text{RMS}{\left(n\right)}^{T}\right]}^{T} \end{array}$$

#### 2.4.2. Baseline ODIN + MorphIC

As described in section 2.3.1, a CNN cannot be efficiently implemented on crossbar cores, which is the architecture ODIN and MorphIC rely on. We will therefore rely solely on fully-connected MLPs networks for both visual and EMG data processing. For the visual input, we used the same subMLP-based network structure as the one described in section 2.3.1, but with gray-scale APS frames. The 40 × 40 cropped APS frames are sub-sampled and fed into four 2-layer subMLPs of architecture 400-210-5, as shown in Figure 3B. The outputs of the four subMLPs are then summed when classifying with a single sensor and are concatenated for the fusion network. The EMG neural network is a 2-layer MLP of architecture 16-230-5. The fusion network is obtained as described above for the Loihi baseline.

#### 2.4.3. Baseline Loihi

As described in section 2.3.2, we used a spiking MLP and a spiking CNN to process and classify DVS events. For the Loihi baseline, we kept the exact same architectures, except for the axonal delays. Moreover, both architectures of the baseline receive the corresponding gray-scale APS frames instead of the DVS events. The baseline MLP architecture and the CNN architectures are shown in Figures 3A,B, respectively. Note that the number of parameters between the baseline networks and the spiking networks implemented on Loihi is slightly different since the input has one channel (gray-scale) in the case of the baseline that uses APS frames while it has two channels (polarity) in the input for Loihi.

The MLP architecture used for the EMG classification is instead composed of two layers of 128 followed by one layer of 5 units. While the input stays of the same size (16) with respect to the network implemented on Loihi, the input features are different since the baseline MLP receives MAV and RMS features while the Loihi receives spikes obtained from the raw signal.

To obtain the fusion network, we eliminate the last layer (classification layer) from both the single sensor networks, concatenate the two penultimate layers of the single sensor networks, and add a common classification layer with five units, one per each class.

#### 2.4.4. Training and Deployment

The models are trained with Keras using Adam optimizer with standard parameters. First, the single modality networks are trained separately, each for 30 epochs. For sensor fusion, output layer retraining is also carried out for 30 epochs. In order to compare the baselines against the neuromorphic systems in terms of energy consumption and inference time, we deployed the baseline models onto the NVIDIA Jetson Nano, an embedded system with a 128-Core Maxwell GPU with 4GB 64-bit LPDDR4 memory 25.6 GB/s^3^.

## 3. Results

Table 1 summarizes the results for Loihi and ODIN+MorphIC with the respective baselines. More details are described in the following sections.

**Table 1:** Comparison of traditional and neuromorphic systems on the task of gesture recognition for both single sensor and sensor fusion.

**System**	**Modality**	**Accuracy (%)**	**Energy (uJ)**	**Inference time (ms)**	EDP (uJ * s)
Spiking CNN (Loihi)	EMG	55.7 ± 2.7	173.2 ± 21.2	5.89 ± 0.18	1.0 ± 0.1
	DVS	92.1 ± 1.2	815.3 ± 115.9	6.64 ± 0.14	5.4 ± 0.8
	EMG+DVS	96.0 ± 0.4	1104.5 ± 58.8	7.75 ± 0.07	8.6 ± 0.5
CNN (GPU)	EMG	68.1 ± 2.8	(25.5 ± 8.4)·10^3^	3.8 ± 0.1	97.3 ± 4.4
	APS	92.4 ± 1.6	(31.7 ± 7.4)·10^3^	5.9 ± 0.1	186.9 ± 3.9
	EMG+APS	95.4 ± 1.7	(32.1 ± 7.9)·10^3^	6.9 ± 0.05	221.1 ± 4.1
Spiking MLP (ODIN + MorphIC)	EMG	53.6 ± 1.4	7.42 ± 0.11	23.5 ± 0.35	0.17 ± 0.01
	DVS	85.1 ± 4.1	57.2 ± 6.8	17.3 ± 2.0	1.00 ± 0.24
	EMG+DVS	89.4 ± 3.0	37.4 ± 4.2	19.5 ± 0.3	0.42 ± 0.08
MLP (GPU)	EMG	67.2 ± 3.6	(23.9 ± 5.6)·10^3^	2.8 ± 0.08	67.2 ± 2.9
	APS	84.2 ± 4.3	(30.2 ± 7.5)·10^3^	6.9 ± 0.1	211.3 ± 6.1
	EMG+APS	88.1 ± 4.1	(32.0 ± 8.9)·10^3^	7.9 ± 0.05	253.0 ± 3.9

*The results of the accuracy are reported with mean and standard deviation obtained over a 3-fold cross validation*.

### 3.1. Loihi Results

The classification performances of these three networks, EMG-Loihi, DVS-Loihi, and Fusion-Loihi, with 3-fold cross-validation and inferenced using 200 *ms* data, are tabulated in Table 2. The core utilization, dynamic power consumption, and inference time in the Loihi hardware are also listed in Table 2. The dynamic power is measured as the difference of total power consumed by the network and the static power when the chip is idle. Since one algorithmic time tick is 1ms long, inference time represents the speedup factor compared to real time.

**Table 2:** Inference statistics of Loihi models on 200 ms-long samples.

**Network**	**Accuracy %**	**Core utilization**	**Dynamic power (mW)**	**Inference speedup**
EMG-Loihi	55.74 ± 2.74	6	29.4± 3.6	(34.01 ± 1.01) ×
DVS-Loihi	92.14 ± 1.23	95	109.0 ± 15.5	(30.14 ± 0.65) ×
Fusion-Loihi	96.04 ± 0.48	100	137.2 ± 7.3	(25.82 ± 0.24) ×

With the spiking MLP implemented on Loihi, we obtained an accuracy of 50.3 ± 1.5, 83.1 ± 3.4, and 83.4 ± 2.1% for the hand-gesture classification task using EMG, DVS and fusion, respectively. Being that these results were significantly worse than the ones obtained with the spiking CNN, we do not report them in Tables 1, 2 and prefer to focus our analysis on the CNN which is better suited for visual tasks. This poor performance is due to temporal resolution of Loihi that causes a drop in the number of spikes in the MLP architecture while this does not happen in the CNN architecture.

The EMG network does not perform as well as in the baseline as shown in Table 1. The reason for this discrepancy can be found in the fact that the baseline method uses EMG from the raw signal of the sensor. However, to process this signal using neuromorphic chips (Loihi and ODIN + MorphIC), the EMG signal is encoded into spikes. With this encoding, part of the information is lost (as is the case for any encoding). Therefore, the baseline method has the advantage of using a signal that has more information and thus it outperforms the neuromorphic approach. Note that these Loihi networks are restricted to 8-bit fixed precision weights and 6-bit fixed precision delays.

**Figure 4:**
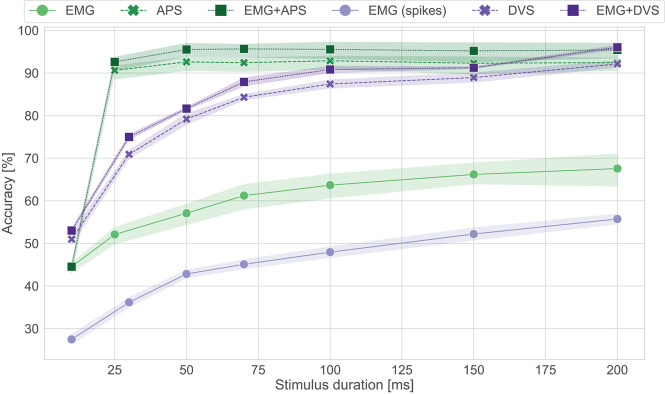
Accuracy vs. stimulus duration for the Loihi system and its software baseline counterpart. In green the results for the CNN (GPU), in purple the results for the spiking CNN (Loihi). No classification is present for APS frames before 25 ms since the frame rate is 20 fps.

To evaluate the performance over time of the Loihi networks, stimulus duration vs. testing accuracy is plotted in Figure 4. We can see that the EMG-Loihi network continues to improve with longer stimulus duration. Table 1 and Figure 4 show the results of the Loihi baseline. From an accuracy point of view the baseline reaches a higher classification accuracy only in the EMG classification, while both the visual classification and fusion are on par with the Loihi networks and show only a non-significant difference. In terms of inference time, the baseline running on the GPU system is systematically faster than Loihi, but never more than 40% faster. As expected, the energy consumption of the GPU system is significantly higher than the Loihi system. Loihi is around 30× more efficient than the baseline with concern to the fusion network and more than 150× and 40× more efficient with concern to the EMG and DVS processing, respectively. Figure 4 shows in more details the effect of stimulus duration on the classification accuracy. As expected, EMG is the modality that suffers more from classification based on short segments (Smith et al., 2011), reaching the best accuracy only after 200 *ms* for both the neuromorphic system and the baseline, while the accuracy for vision and fusion modalities saturate much more quickly, in around 100 ms for the neuromorphic system and 50 ms for the baseline. The traditional system reaches its best performance after 50 ms while the neuromorphic system reaches its best performance after 200*ms*. One should, however, also note that the DVS sensor contains only the edge information of the scene whereas the baseline network uses the image frame. Therefore, the spiking CNN requires some time to integrate the input information from DVS. Despite the inherent delays in a spiking CNN, the Loihi CNN can respond to the input within a few ms of inputs. However, for the vision modality, notice that, because the frame rate of the camera is 20 fps, there is no classification before 25*ms*. Therefore, for short stimulus duration, the neuromorphic system has higher accuracy than the traditional system.

### 3.2. ODIN + MorphIC Results

Inference statistics for a 200 ms sample duration are reported in Table 3 for the EMG-ODIN, DVS-MorphIC, and Fusion-ODIN networks. Chip utilization is computed as the percentage of neuron resources taken by the hidden and output layers in ODIN and MorphIC, while the power consumption *P* of the crossbar cores of both chips can be decomposed as

$$\begin{array}{l} P=P_{\text{leak}}+P_{\text{idle}}f_{\text{clk}}+E_{\text{SOP}}r_{\text{SOP}}, \end{array}$$

where *P*_leak_ is the chip leakage power and *P*_leak_ + *P*_idle_*f*_clk_ represents the static power consumption when a clock of frequency *f*_clk_ is connected, without network activity. The term *E*_SOP_*r*_SOP_ thus represents the dynamic power consumption, where *E*_SOP_ is the energy per synaptic operation (SOP) and *r*_SOP_ is the SOP processing rate, each SOP taking two clock cycles. Detailed power models extracted from chip measurements of ODIN and MorphIC are provided in Frenkel et al. (2019a,b), respectively. The results reported in Tables 1, 3 are obtained with ODIN and MorphIC optimizing for power, under the conditions summarized in Table 4. The dynamic power consumption reported in Table 4 reflects the regime in which ODIN and the four cores of MorphIC run at the maximum SOP processing rate *r*_SOP_ = *f*_clk_/2.

**Table 3:** Inference statistics of ODIN and MorphIC models on 200 ms-long samples.

**Network**	**Accuracy (%)**	**Chip utilization (%)**		**Dyn. power (mW)**		**Processing time (ms)**		**Inference speedup**
		**ODIN**	**MorphIC**	**ODIN**	**MorphIC**	**ODIN**	**MorphIC**	
EMG-ODIN	53.65 ± 1.37	91.8	–	0.315	–	23.5	–	8.5 ×
DVS-MorphIC	85.17 ± 4.11	–	42.0	–	3.3	–	17.3	11.6 ×
Fusion-ODIN	89.44 ± 3.02	91.8	41.0	0.315	3.3	19.5	9.5	10.3 ×

**Table 4:** Low-power operating conditions of ODIN and MorphIC at minimum supply voltage.

**Chip**	**Supply voltage (V)**	***E*_SOP_ (pJ)**	***Max. f*_clk_ (MHz)**
ODIN	0.55	8.4	75
MorphIC	0.8	30	55

A limitation of the crossbar-based architecture of ODIN and MorphIC is that each neuron spike leads to a systematic processing of all neurons in the core, thus potentially leading to a significant amount of dummy operations (Frenkel et al., 2019b). Taking the example of the DVS-MorphIC network with a crossbar core of 512 neurons (Figure 3B), each input spike leads to 512 SOPs, of which only 210 are useful for hidden layer processing. Similarly, each spike from a hidden layer neuron leads to 512 SOPs, of which only five are actually used for output layer processing. The induced overhead is thus particularly critical for output layer processing, which degrades both the energy per inference and the inference time^4^. However, this problem is partly mitigated in the Fusion-ODIN network for output layer processing. Indeed, when resorting to an external mapping table (section 2.3.1), hidden layer spikes can be remapped back to the sensor fusion output layer of ODIN with specific single-SOP AER events (Frenkel et al., 2019a), thus avoiding the dummy SOP overhead and leading to a lower energy and inference time compared to the standalone EMG-ODIN and DVS-MorphIC networks (Tables 1, 3). As described in section 2.3.1, the fusion results exclude the mapping table overhead.

**Figure 5:**
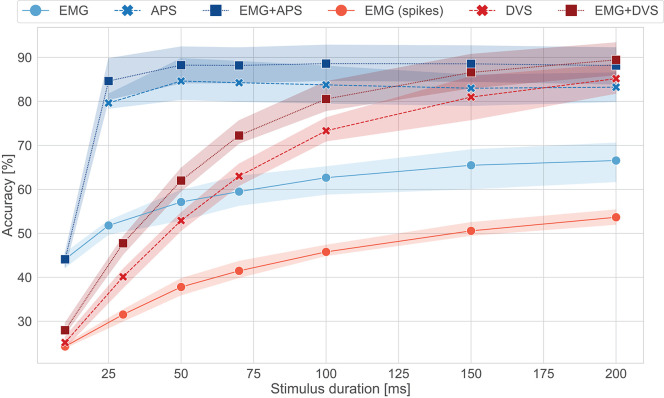
Accuracy vs. stimulus duration for the ODIN + MorphIC system and its software baseline counterpart. In blue the results for the MLP (GPU), in red the results for the spiking MLP (ODIN + MorphIC). No classification is present for APS frames before 25 ms since the frame rate is 20 fps.

The comparison of the results obtained with ODIN + MorphIC to those obtained with its GPU baseline counterpart (Table 1 and Figure 5) leads to conclusions similar to those already drawn with Loihi in section 3.1, with the difference that while the GPU system is significantly faster, between 2× and 10× faster, the ODIN + MorphIC neuromorphic system is between 500× and 3,200× more energy-efficient. Moreover, it appears from Figure 5 that the EMG-ODIN, DVS-MorphIC and Fusion-ODIN networks basically perform at chance level for a 10-ms stimulus duration. This comes from the fact that the firing thresholds of the networks were selected based on a 200-ms stimulus duration, which leads the output neurons to remain silent and never cross their firing threshold when insufficient input spike data is provided. This problem could be alleviated by reducing the neuron firing thresholds for shorter stimulus durations.

### 3.3. EDP and Computational Complexity

**Figure 6:**
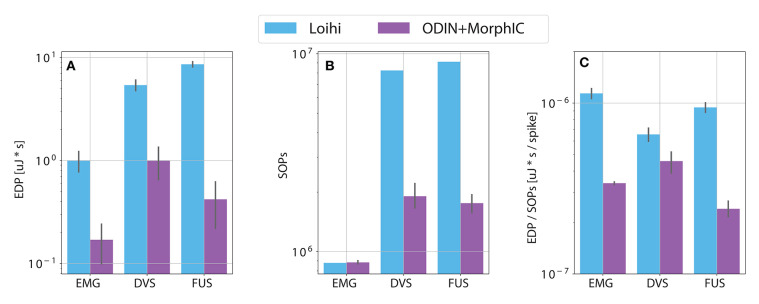
Comparison between the two neuromorphic system with respect to **(A)** energy delay product (EDP) (see section 1), **(B)** number of synaptic operations (SOPs) (see section 2.3.1), **(C)** EDP normalized by the number of SOPs.

Figure 6 shows a comparison between the Loihi system and the ODIN + MorphIC system in terms of EDP, number of operations per classification and a ratio between these two quantities. While panel (a) reports the same numbers as in Table 1, panels (b) and (c) allow for a more fair comparison of energy consumption between the two neuromorphic systems. From panel (b), we can see how the number of operations is similar for the EMG networks, both being MLPs for the two neuromorphic systems. Differently, the number of operations for the visual input and the fusion differ substantially between the two systems due to the use of a CNN in the Loihi system. Taking this into account, we can see in panel (c) that the normalized energy consumption tends to be similar for both systems, more than the EDP in panel (a) is.

## 4. Discussions

As it has been discussed in Davies (2019), there is a real need for a benchmark in the neuromorphic engineering field to compare the metrics of accuracy, energy, and latency. ML benchmarks, such as ImageNet for image classification (Deng et al., 2009), Chime challenges for speech recognition (Barker et al., 2015), and the Ninapro dataset containing kinematic and surface EMG for prosthetic applications (Atzori et al., 2014) are not ideal for neuromorphic chips as they require high performance computing for processing. For example, floating point bit resolution, large amounts of data and large power consumption. There have been some efforts in creating relevant event-based datasets, such as N-MNIST (Orchard et al., 2015), the spiking version of the widespread MNIST digits recognition dataset, N-TIDIGITS18 (Anumula et al., 2018), the spiking version of the spoken digits recognition dataset from LDC TIDIGITS, and the DVS gesture recognition dataset from IBM (Amir et al., 2017). These datasets are either toy examples or are not meant for real-world applications. Here, we are introducing a hand gesture benchmark in English sign language (e.g., ILY) using the DVS and Myo sensors. This kind of benchmark can be directly used as a preliminary test for Brain-Machine Interface (BMI)/personalized medicine applications. We have collected this dataset from 21 people and in this paper have benchmarked it on three digital neuromorphic chips, measuring the accuracy, energy, and inference time. We believe this work takes an important first step in the direction of a real use-case (e.g., rehabilitation, sports applications, and sign interpretation) which we would like to encourage the community to use.

Although the dataset we provided is on static gestures, the DVS and the spiking EMG signals provide the capability for low-power processing using event-based neuromorphic chips and enable embedded systems with online on-site processing without having to send the data to remote sensors. Therefore, this work is an important first step toward edge-computing applications. The static dataset also helps with reducing the noise from the EMG signals as we mentioned in section 2.2. However, this does not move away from the real application as we have shown in a live demo in Ceolini et al. (2019a).

The selected multi-sensor data fusion, which combines vision and EMG sensors, derives from the need of multiple sources to help the classification in real-scenario cases. Although the results show a small improvement due to the EMG sensors, they still provide some classification in case light conditions or camera occlusions are not ideal. In addition, for specific applications, such as neuroprosthetic control, the EMG is integrated in the prosthetic device and, eventually, the camera can act as a support input helping during calibration or more advanced tasks, such as sensory-motor closed loop (Jiang et al., 2012).

Since the event-based neuromorphic chips require inputs in the form of events, the continuous sensory signals have to be encoded into spikes for an event-driven processing. This quantization loses information (and hence accuracy) in comparison to the analog information processing in trade-off with the low power consumption of event-based systems which is required for edge computing. To compensate for the loss of information and accuracy, it is important to merge information from multiple sensors in a sensory fusion setup. In this setting, the information loss by quantization from one sensor can be made up for by another one. This is similar to how humans and animals perceive their environment through diverse sensory channels: vision, audition, touch, smell, proprioception, etc. From a biological perspective, the fundamental reason lies in the concept of degeneracy in neural structures (Edelman, 1987), which means that any single function can be carried out by more than one configuration of neural signals, so that the biological system still functions with the loss of one component. It also means that sensory systems can educate each other, without an external teacher (Smith and Gasser, 2005). The same principles can be applied for artificial systems, as information about the same phenomenon in the environment can be acquired from various types of sensors: cameras, microphones, accelerometers, etc. Each sensory-information can be considered as a modality. Due to the rich characteristics of natural phenomena, it is rare that a single modality provides a complete representation of the phenomenon of interest (Lahat et al., 2015).

There are mainly two strategies for multi-modal fusion in the literature (Cholet et al., 2019): (1) data-level fusion (early fusion) where modalities are concatenated then learned by a unique model, and (2) score-level fusion (late fusion) where modalities are learned by distinct models and only after their predictions are fused with another model that provides a final decision. Early fusion, including feature-level fusion, suffers from a compatibility problem (Peng et al., 2016) and does not generalize well. Additionally, neural-based early fusion increases the memory footprint and the computational cost of the process, by inducing a full connectivity at the first classification stages. It is an important factor to take into consideration when choosing a fusion strategy (Castanedo, 2013), especially for embedded systems. Therefore, we follow a late fusion approach with a classifier-level fusion, which has been shown to perform better than feature-level fusion for classification tasks (Guo et al., 2014; Peng et al., 2016; Biagetti et al., 2018). It is close to score-level fusion by combining the penultimate layers of the base (unimodal) classifiers in a meta-level (multimodal) classifier that uses the natural complementarity of different modalities to improve the overall classification accuracy.

In this context, to have a fair comparison, the central question is the difference between the completely traditional approaches, such as the CNN and MLP baselines, vs. the event-based neuromorphic one. In the baseline, the EMG features are manually extracted, and the classification is done on the extracted features. Note that this pipeline is completely different from the event-based neuromorphic approach which extracts the features directly from the events. Another important thing to mention here is that although we have encoded the signals separately, this sensory information can be directly encoded to events at the front-end. This has already been established for audio and visual sensors (Lichtsteiner et al., 2006; Chan et al., 2007) and there have also recently been design efforts for other signals such the biomedical ones (Corradi and Indiveri, 2015).

To have a reference point for comparison, we trained the same network architecture used for the two neuromorphic setups. As can be seen in Table 1, the baseline accuracy on the fusion is on par with both Loihi and ODIN + MorphIC, despite the lower bit resolution on the neuromorphic chips in comparison with the 32-bit floating point resolutions on GPU in the baseline approach. We speculate that this is because the SLAYER training model already takes into account the low bit precision and thus calculates the gradients, respectively. Similar to that, ODIN and MorphIC take a quantization-aware training approach which calculates the weights based on the available on-chip precision. As can be seen from all the experiments in Table 1, the classification accuracy using only the EMG sensor is relatively low. However, it should be noted that this is the result of having a model which is trained across subjects and there are multiple sources of variability across subjects: (i) The placement of the EMG sensor is not necessarily in the same position (with respect to the forearm muscles) for every subject. (ii) Every subject performs the gestures in a unique manner. (iii) The muscle strength is different for every subject. In addition, since the EMG is directly measured from surface electrodes, it acquires noise while traveling through the skin, background noise from electronics, ambient noise, and so forth. In a real-world application, the network model can be trained on a single subject's data, yielding much higher accuracy. Moreover, having the online learning abilities on the neuromorphic chip can aid in adapting these models to every subject uniquely. Such online learning modules already exist in Loihi as well as in ODIN and MorphIC, which can be exploited in the future to boost the classification accuracy of EMG signals. Furthermore, it becomes apparent that the fusion accuracy is close, if not higher, at about 4% to the accuracy achieved with the DVS single sensor. However, the importance of the EMG signal is in the wearable application since it is a natural way to control prosthesis and it is a direct measure of the activity and movement in the muscles. Given the noisy nature of the EMG signal, it is critical to combine it with the visual input to boost the accuracy. But even given the noisy nature of the signal, it still allows to retrieve relevant information which helps boosting the accuracy of the fusion.

It is worth noting that while the accuracy between the spiking MLP on Loihi and ODIN + MorphIC are directly comparable, the results regarding the spiking CNN on Loihi and the spiking MLP on ODIN + MorphIC are not. This is because the two architectures use different features and resources on their respective neuromorphic systems (as already described in section 2.3). Based on this, there are different constraints present in the two chips. Traditionally, a CNN architecture is used for image classification which is the network we used on the Loihi chip, given the large number of neurons that are available (128k) on this general-purpose platform. However, since ODIN and MorphIC are small-scale devices compared to Loihi, the number of neurons are a lot more constrained (i.e., 256 neurons for ODIN, 2k for MorphIC). Therefore, we resorted to using a fully-connected MLP topology instead of a CNN for image classification in MorphIC.

Regarding the latency, it is important to mention that for real-world prosthetic applications, the latency budget is below 250 ms (Smith et al., 2011). This means that if the processing happens within this budget, the patient will not feel the lag of the system. Hence, optimizing the system for having lower latency than 200 ms will not be beneficial as the patient will not feel the latency below 200 ms. Therefore, within this budget, other parameters can be optimized. The neuromorphic approach is very advantageous in this case since it trades-off power with latency, but it stays within the latency budget that is required. Contrarily, the GPU system has an overall faster inference time but uses much more energy. It is worth mentioning that our results are reported in accelerated time, however, the EMG and DVS are slowly changing signals, and thus, even though the classification is done very fast, the system has to wait for the inputs to arrive. Therefore, it is as if the system is being run in real-time. Here, there is a trade-off between the memory that is storing the streaming data for processing and the dynamic energy consumption. The accelerated time allows for lower energy consumption as the system is on for a shorter time, however, this comes with the caveat that the input has to be buffered for at least 200 ms in off-chip memory, therefore inducing a power and resource overhead.

The final comparison provided by Figure 6 shows how the two systems have a similar energy consumption when this is normalized by the number of operations done to run the network and obtain one classification output. While ODIN + MorphIC consumes less per classification in absolute terms, when considering the number of operations, it performs comparably to Loihi. When deploying a neuromorphic system, one has to take into account all these aspects. Meaning not only is there a trade-off between speed and energy consumption but there is also one between accuracy and energy consumption, given the fact that a more complex network architecture may have more predictive power while having a higher energy demand. Overall, one has to look for the best trade-off in the context of a particular application, the malleability of neuromorphic hardware enables this adaptation to the task-dependent constraints within a framework of state of the art results with respect to system performance.

## Data Availability Statement

The datasets analyzed for this study can be found in the Zenodo, open access repository, http://doi.org/10.5281/zenodo.3663616. All the code used for the reported experiments can be found at https://github.com/Enny1991/dvs_emg_fusion.

## Author Contributions

EC, CF, and SS contributed equally to the work. EC, GT, MP, and ED participated equally to the development of the work idea and collected the dataset. EC and LK were responsible for the baseline experiments. CF and SS implemented the ODIN + MorphIC and Loihi pipelines, respectively. SS implemented the SLAYER framework and adapted it for the specific application. All authors contributed to the writing of the paper.

## Conflict of Interest

The authors declare that the research was conducted in the absence of any commercial or financial relationships that could be construed as a potential conflict of interest.

## Footnotes

^1^The HDL source code and documentation of ODIN are publicly available at https://github.com/ChFrenkel/ODIN.

^2^SLAYER-PyTorch is publicly available at https://github.com/bamsumit/slayerPytorch.

^3^https://developer.nvidia.com/embedded/jetson-nano-developer-kit

^4^As discussed in (Frenkel et al., 2019b), a simple extension providing post-synaptic start and end addresses would avoid these dummy SOPs and allow for an efficient processing of fully-connected layers, which will be included in future generations of the chips.

## References

[B1] Amir A. Taba B. Berg D. Melano T. McKinstry J. Nolfo C. D. (2017). “A low power, fully event-based gesture recognition system,” in 2017 IEEE Conference on Computer Vision and Pattern Recognition (CVPR) (Honolulu, HI), 7388–7397. 10.1109/CVPR.2017.781

[B2] Anumula J. Neil D. Delbruck T. Liu S.-C. (2018). Feature representations for neuromorphic audio spike streams. Front. Neurosci. 12:23. 10.3389/fnins.2018.00023 29479300 PMC5811520

[B3] Atzori M. Gijsberts A. Castellini C. Caputo B. Hager A.-G. M. Elsig S.. (2014). Electromyography data for non-invasive naturally-controlled robotic hand prostheses. Sci. Data 1:140053. 10.1038/sdata.2014.53 25977804 PMC4421935

[B4] Barker J. Marxer R. Vincent E. Watanabe S. (2015). “The third ‘chime’ speech separation and recognition challenge: dataset, task and baselines,” in 2015 IEEE Workshop on Automatic Speech Recognition and Understanding (ASRU) (Scottsdale, AZ), 504–511. 10.1109/ASRU.2015.7404837

[B5] Benatti S. Casamassima F. Milosevic B. Farella E. Schönle P. Fateh S.. (2015). A versatile embedded platform for emg acquisition and gesture recognition. IEEE Trans. Biomed. Circuits Syst. 9, 620–630. 10.1109/TBCAS.2015.2476555 26513799

[B6] Benjamin B. V. Gao P. McQuinn E. Choudhary S. Chandrasekaran A. R. Bussat J.-M. (2014). Neurogrid: a mixed-analog-digital multichip system for large-scale neural simulations. Proc. IEEE 102, 699–716. 10.1109/JPROC.2014.2313565

[B7] Biagetti G. Crippa P. Falaschetti L. (2018). Classifier level fusion of accelerometer and semg signals for automatic fitness activity diarization. Sensors 18:2850. 10.3390/s18092850 30158443 PMC6164365

[B8] Brader J. M. Senn W. Fusi S. (2007). Learning real-world stimuli in a neural network with spike-driven synaptic dynamics. Neural Comput. 19, 2881–2912. 10.1162/neco.2007.19.11.2881 17883345

[B9] Braun S. Neil D. Anumula J. Ceolini E. Liu S. (2019). “Attention-driven multi-sensor selection,” in 2019 International Joint Conference on Neural Networks (IJCNN) (Budapest), 1–8. 10.1109/IJCNN.2019.8852396

[B10] Castanedo F. (2013). A review of data fusion techniques. TheScientificWorldJournal 2013:704504. 10.1155/2013/704504 24288502 PMC3826336

[B11] Ceolini E. Taverni G. Khacef L. Payvand M. Donati E. (2019a). “Live demostration: sensor fusion using emg and vision for hand gesture classification in mobile applications,” in 2019 IEEE Biomedical Circuits and Systems Conference (BioCAS) (Nara), 1 10.1109/BIOCAS.2019.8919163

[B12] Ceolini E. Taverni G. Khacef L. Payvand M. Donati E. (2019b). Sensor fusion using EMG and vision for hand gesture classification in mobile applications. arXiv 1910.11126 10.1109/BIOCAS.2019.8919210

[B13] Chan V. Liu S.-C. van Schaik A. (2007). Aer ear: A matched silicon cochlea pair with address event representation interface. IEEE Trans. Circuits Syst. I Reg. Pap. 54, 48–59. 10.1109/TCSI.2006.887979

[B14] Chen C. Yu Y. Ma S. Sheng X. Lin C. Farina D. (2020). Hand gesture recognition based on motor unit spike trains decoded from high-density electromyography. Biomed. Signal Process. Control 55:101637 10.1016/j.bspc.2019.101637

[B15] Cheok M. J. Omar Z. Jaward M. H. (2019). A review of hand gesture and sign language recognition techniques. Int. J. Mach. Learn. Cybern. 10, 131–153. 10.1007/s13042-017-0705-5

[B16] Cholet S. Paugam-Moisy H. Regis S. (2019). “Bidirectional associative memory for multimodal fusion: a depression evaluation case study,” in 2019 International Joint Conference on Neural Networks (IJCNN) (Budapest), 1–6. 10.1109/IJCNN.2019.8852089

[B17] Cicirelli G. Attolico C. Guaragnella C. D'Orazio T. (2015). A kinect-based gesture recognition approach for a natural human robot interface. Int. J. Adv. Robot. Syst. 12:22 10.5772/59974

[B18] Corradi F. Indiveri G. (2015). A neuromorphic event-based neural recording system for smart brain-machine-interfaces. IEEE Trans. Biomed. Circuits Syst. 9, 699–709. 10.1109/TBCAS.2015.2479256 26513801

[B19] Courbariaux M. Bengio Y. David J.-P. (2015). “Binaryconnect: training deep neural networks with binary weights during propagations,” in Advances in Neural Information Processing Systems, eds Cortes C. Lawrence N. D. Lee D. D. Sugiyama M. Garnett R. (Montreal, QC: Curran Associates, Inc.), 3123–3131.

[B20] Davies M. (2019). Benchmarks for progress in neuromorphic computing. Nat. Mach. Intell. 1, 386–388. 10.1038/s42256-019-0097-1

[B21] Davies M. Srinivasa N. Lin T.-H. Chinya G. Cao Y. Choday S. H. (2018). Loihi: a neuromorphic manycore processor with on-chip learning. IEEE Micro 38, 82–99. 10.1109/MM.2018.112130359

[B22] Debes C. Merentitis A. Heremans R. Hahn J. Frangiadakis N. van Kasteren T. (2014). Hyperspectral and LiDAR data fusion: outcome of the 2013 grss data fusion contest. IEEE J. Select. Top. Appl. Earth Observ. Rem. Sens. 7, 2405–2418. 10.1109/JSTARS.2014.2305441

[B23] Deiss S. R. Douglas R. J. Whatley A. M. (1999). “A pulse-coded communications infrastructure for neuromorphic systems,” in Pulsed Neural Networks, eds Maass W. Bishop C. M. (Cambridge, MA: MIT Press), 157–178.

[B24] Deng J. Dong W. Socher R. Li L.-J. Li K. Fei-Fei L. (2009). “Imagenet: a large-scale hierarchical image database,” in 2009 IEEE Conference on Computer Vision and Pattern Recognition (Miami, FL: IEEE), 248–255. 10.1109/CVPR.2009.5206848

[B25] Donati E. Payvand M. Risi N. Krause R. Burelo K. Indiveri G. (2018). Processing EMG signals using reservoir computing on an event-based neuromorphic system. in 2018 IEEE Biomedical Circuits and Systems Conference (BioCAS), pages 1–4. IEEE 10.1109/BIOCAS.2018.8584674

[B26] Donati E. Payvand M. Risi N. Krause R. B. Indiveri G. (2019). Discrimination of EMG signals using a neuromorphic implementation of a spiking neural network. IEEE Trans. Biomed. Circuits Syst. 13, 795–803. 10.1109/TBCAS.2019.2925454 31251192

[B27] Došen S. Cipriani C. Kostić M. Controzzi M. Carrozza M. C. Popović D. B. (2010). Cognitive vision system for control of dexterous prosthetic hands: experimental evaluation. J. Neuroeng. Rehabil. 7:42. 10.1186/1743-0003-7-42 20731834 PMC2940869

[B28] Droniou A. Ivaldi S. Sigaud O. (2015). Deep unsupervised network for multimodal perception, representation and classification. Robot. Auton. Syst. 71, 83–98. 10.1016/j.robot.2014.11.005

[B29] Edelman G. M. (1987). Neural Darwinism: The Theory of Neuronal Group Selection. New York, NY: Basic Books. 10.1126/science.240.4860.1802 17842436

[B30] Frenkel C. Lefebvre M. Legat J.-D. Bol D. (2019a). A 0.086-mm^2^ 12.7-pj/sop 64k-synapse 256-neuron online-learning digital spiking neuromorphic processor in 28-nm CMOS. IEEE Trans. Biomed. Circuits Syst. 13, 145–158. 10.1109/TBCAS.2018.2880425 30418919

[B31] Frenkel C. Legat J.-D. Bol D. (2019b). Morphic: a 65-nm 738k-synapse/mm^2^ quad-core binary-weight digital neuromorphic processor with stochastic spike-driven online learning. IEEE Trans. Biomed. Circuits Syst. 13, 999–1010. 10.1109/TBCAS.2019.2928793 31329562

[B32] Furber S. B. Galluppi F. Temple S. Plana L. A. (2014). The spinnaker project. Proc. IEEE 102, 652–665. 10.1109/JPROC.2014.2304638

[B33] Gerstner W. (1995). Time structure of the activity in neural network models. Phys. Rev. E 51, 738–758. 10.1103/PhysRevE.51.738 9962697

[B34] Ghazaei G. Alameer A. Degenaar P. Morgan G. Nazarpour K. (2017). Deep learning-based artificial vision for grasp classification in myoelectric hands. J. Neural Eng. 14:036025. 10.1088/1741-2552/aa6802 28467317

[B35] Gigli A. Gregori V. Cognolato M. Atzori M. Gijsberts A. (2018). “Visual cues to improve myoelectric control of upper limb prostheses,” in 2018 7th IEEE International Conference on Biomedical Robotics and Biomechatronics (Biorob) (Enschede: IEEE), 783–788. 10.1109/BIOROB.2018.8487923

[B36] Guo H. Chen L. Shen Y. Chen G. (2014). “Activity recognition exploiting classifier level fusion of acceleration and physiological signals,” in UbiComp 2014–Adjunct Proceedings of the 2014 ACM International Joint Conference on Pervasive and Ubiquitous Computing (Seattle, WA), 63–66. 10.1145/2638728.2638777

[B37] Haria A. Subramanian A. Asokkumar N. Poddar S. Nayak J. S. (2017). Hand gesture recognition for human computer interaction. Proc. Comput. Sci. 115, 367–374. 10.1016/j.procs.2017.09.092

[B38] Hoeks C. Barentsz J. Hambrock T. Yakar D. Somford D. Heijmink S.. (2011). Prostate cancer: multiparametric MR imaging for detection, localization, and staging. Radiology 261, 46–66. 10.1148/radiol.11091822 21931141

[B39] Horwitz B. Poeppel D. (2002). How can EEG/MEG and fMRI/PET data be combined? Hum. Brain Mapp. 17, 1–3. 10.1002/hbm.10057 12203682 PMC6871863

[B40] Hubara I. Courbariaux M. Soudry D. El-Yaniv R. Bengio Y. (2016). “Binarized neural networks,” in Advances in Neural Information Processing Systems, eds Lee D. D. Sugiyama M. Luxburg U. V. Guyon I. Garnett R. (Barcelona: Curran Associates, Inc.), 4107–4115.

[B41] Hubara I. Courbariaux M. Soudry D. El-Yaniv R. Bengio Y. (2017). Quantized neural networks: training neural networks with low precision weights and activations. J. Mach. Learn. Res. 18, 6869–6898. 10.5555/3122009.3242044

[B42] Izhikevich E. M. (2004). Which model to use for cortical spiking neurons? IEEE Trans. Neural Netw. 15, 1063–1070. 10.1109/TNN.2004.832719 15484883

[B43] Jiang N. Dosen S. Muller K.-R. Farina D. (2012). Myoelectric control of artificial limbs—is there a need to change focus? IEEE Signal Process. Mag. 29, 152–150. 10.1109/MSP.2012.2203480

[B44] Lahat D. Adali T. Jutten C. (2015). Multimodal data fusion: an overview of methods, challenges, and prospects. Proc. IEEE 103, 1449–1477. 10.1109/JPROC.2015.2460697

[B45] Lee H.-Y. Hsu C.-M. Huang S.-C. Shih Y.-W. Luo C.-H. (2005). Designing low power of sigma delta modulator for biomedical application. Biomed. Eng. Appl. Basis Commun. 17, 181–185. 10.4015/S1016237205000287

[B46] Lichtsteiner P. Posch C. Delbruck T. (2006). “A 128 × 128 120 dB 30 MW asynchronous vision sensor that responds to relative intensity change,” in 2006 IEEE International Solid State Circuits Conference-Digest of Technical Papers (San Francisco, CA: IEEE), 2060–2069. 10.1109/ISSCC.2006.1696265

[B47] Liu H. Wang L. (2018). Gesture recognition for human-robot collaboration: a review. Int. J. Ind. Ergon. 68, 355–367. 10.1016/j.ergon.2017.02.004

[B48] Liu S.-C. Delbruck T. Indiveri G. Whatley A. Douglas R. (2014). Event-Based Neuromorphic Systems. Hoboken, NJ: John Wiley & Sons.

[B49] Loss J. F. Cantergi D. Krumholz F. M. La Torre M. Candotti C. T. (2012). “Evaluating the electromyographical signal during symmetrical load lifting,” in Applications of EMG in Clinical and Sports Medicine, ed Steele C. (Norderstedt: Books on Demand), 1.

[B50] Markovic M. Dosen S. Cipriani C. Popovic D. Farina D. (2014). Stereovision and augmented reality for closed-loop control of grasping in hand prostheses. J. Neural Eng. 11:046001. 10.1088/1741-2560/11/4/046001 24891493

[B51] Markovic M. Dosen S. Popovic D. Graimann B. Farina D. (2015). Sensor fusion and computer vision for context-aware control of a multi degree-of-freedom prosthesis. J. Neural Eng. 12:066022. 10.1088/1741-2560/12/6/066022 26529274

[B52] Meier K. (2015). “A mixed-signal universal neuromorphic computing system,” in 2015 IEEE International Electron Devices Meeting (IEDM) (Washington, DC: IEEE), 4–6. 10.1109/IEDM.2015.7409627

[B53] Merolla P. A. Arthur J. V. Alvarez-Icaza R. Cassidy A. S. Sawada J. Akopyan F.. (2014). A million spiking-neuron integrated circuit with a scalable communication network and interface. Science 345, 668–673. 10.1126/science.1254642 25104385

[B54] Moons B. Goetschalckx K. Van Berckelaer N. Verhelst M. (2017). “Minimum energy quantized neural networks,” in 2017 51st Asilomar Conference on Signals, Systems, and Computers (Pacific Grove, CA: IEEE), 1921–1925. 10.1109/ACSSC.2017.8335699

[B55] Moradi S. Qiao N. Stefanini F. Indiveri G. (2017). A scalable multicore architecture with heterogeneous memory structures for dynamic neuromorphic asynchronous processors (DYNAPs). IEEE Trans. Biomed. Circuits Syst. 12, 106–122. 10.1109/TBCAS.2017.2759700 29377800

[B56] Neckar A. Fok S. Benjamin B. V. Stewart T. C. Oza N. N. Voelker A. R. (2018). Braindrop: a mixed-signal neuromorphic architecture with a dynamical systems-based programming model. Proc. IEEE 107, 144–164. 10.1109/JPROC.2018.2881432

[B57] Neftci E. Mostafa H. Zenke F. (2019). Surrogate gradient learning in spiking neural networks. arXiv abs/1901.09948.

[B58] Orchard G. Jayawant A. Cohen G. K. Thakor N. (2015). Converting static image datasets to spiking neuromorphic datasets using saccades. Front. Neurosci. 9:437 10.3389/fnins.2015.00437 26635513 PMC4644806

[B59] Paszke A. Gross S. Chintala S. Chanan G. Yang E. DeVito Z. (2017). “Automatic differentiation in PyTorch,” in NeurIPS Autodiff Workshop (Long Beach, CA).

[B60] Peng L. Chen L. Wu X. Guo H. Chen G. (2016). Hierarchical complex activity representation and recognition using topic model and classifier level fusion. IEEE Trans. Biomed. Eng. 64, 1369–1379. 10.1109/TBME.2016.2604856 28113223

[B61] Phinyomark A. N. Khushaba R. Scheme E. (2018). Feature extraction and selection for myoelectric control based on wearable EMG sensors. Sensors 18:1615. 10.3390/s18051615 29783659 PMC5982518

[B62] Pitti A. Blanchard A. Cardinaux M. Gaussier P. (2012). “Gain-field modulation mechanism in multimodal networks for spatial perception,” in 2012 12th IEEE-RAS International Conference on Humanoid Robots (Humanoids 2012) (Osaka), 297–302. 10.1109/HUMANOIDS.2012.6651535

[B63] Qiao N. Mostafa H. Corradi F. Osswald M. Stefanini F. Sumislawska D.. (2015). A reconfigurable on-line learning spiking neuromorphic processor comprising 256 neurons and 128k synapses. Front. Neurosci. 9:141. 10.3389/fnins.2015.00141 25972778 PMC4413675

[B64] Rivet B. Wang W. Naqvi S. M. Chambers J. A. (2014). Audiovisual speech source separation: an overview of key methodologies. IEEE Signal Process. Mag. 31, 125–134. 10.1109/MSP.2013.2296173

[B65] Rueckauer B. Lungu I.-A. Hu Y. Pfeiffer M. Liu S.-C. (2017). Conversion of continuous-valued deep networks to efficient event-driven networks for image classification. Front. Neurosci. 11:682. 10.3389/fnins.2017.00682 29375284 PMC5770641

[B66] Shivappa S. T. Trivedi M. M. Rao B. D. (2010). Audiovisual information fusion in human–computer interfaces and intelligent environments: a survey. Proc. IEEE 98, 1692–1715. 10.1109/JPROC.2010.2057231

[B67] Shrestha S. B. Orchard G. (2018). “SLAYER: spike layer error reassignment in time,” in Advances in Neural Information Processing Systems 31, eds Bengio S. Wallach H. Larochelle H. Grauman K. Cesa-Bianchi N. Garnett R. (Montreal, QC: Curran Associates, Inc.), 1419–1428.

[B68] Smith L. Gasser M. (2005). The development of embodied cognition: six lessons from babies. Artif. Life 11, 13–29. 10.1162/1064546053278973 15811218

[B69] Smith L. H. Hargrove L. J. Lock B. A. Kuiken T. A. (2011). Determining the optimal window length for pattern recognition-based myoelectric control: balancing the competing effects of classification error and controller delay. IEEE Trans. Neural Syst. Rehabil. Eng. 19, 186–192. 10.1109/TNSRE.2010.2100828 21193383 PMC4241762

[B70] Tan A.-H. Subagdja B. Wang D. Meng L. (2019). Self-organizing neural networks for universal learning and multimodal memory encoding. Neural Netw. 120, 58–73. 10.1016/j.neunet.2019.08.020 31537437

[B71] Thakur C. S. Molin J. L. Cauwenberghs G. Indiveri G. Kumar K. Qiao N. (2018). Large-scale neuromorphic spiking array processors: a quest to mimic the brain. Front. Neurosci. 12:891 10.3389/fnins.2018.00891 30559644 PMC6287454

[B72] Turk M. (2014). Multimodal interaction: a review. Pattern Recogn. Lett. 36, 189–195. 10.1016/j.patrec.2013.07.003

[B73] Yasen M. Jusoh S. (2019). A systematic review on hand gesture recognition techniques, challenges and applications. PeerJ Comput. Sci. 5:e218 10.7717/peerj-cs.218 PMC7924500 33816871

[B74] Zahra O. Navarro-Alarcon D. (2019). “A self-organizing network with varying density structure for characterizing sensorimotor transformations in robotic systems,” in Towards Autonomous Robotic Systems, eds Althoefer K. Konstantinova J. Zhang K. (Cham: Springer International Publishing), 167–178. 10.1007/978-3-030-25332-5_15

[B75] Zenke F. Ganguli S. (2018). SuperSpike: supervised learning in multilayer spiking neural networks. Neural Comput. 30, 1514–1541. 10.1162/neco_a_01086 29652587 PMC6118408

[B76] Zhang Y. Wang Z. Du J. (2019). “Deep fusion: an attention guided factorized bilinear pooling for audio-video emotion recognition,” in 2019 International Joint Conference on Neural Networks (IJCNN) (Budapest), 1–8. 10.1109/IJCNN.2019.8851942

[B77] Zhao D. Zeng Y. (2019). “Dynamic fusion of convolutional features based on spatial and temporal attention for visual tracking,” in 2019 International Joint Conference on Neural Networks (IJCNN) (Budapest), 1–8. 10.1109/IJCNN.2019.8852301

